# Auxiliary Sensor-Based Borehole Transient Electromagnetic System for the Nondestructive Inspection of Multipipe Strings

**DOI:** 10.3390/s17081836

**Published:** 2017-08-09

**Authors:** Bo Dang, Ling Yang, Na Du, Changzan Liu, Ruirong Dang, Bin Wang, Yan Xie

**Affiliations:** 1 Key Laboratory of Education Ministry for Photoelectric Logging and Detecting of Oil and Gas, Xi’an Shiyou University, Xi’an 710065, China; lingyang2915@163.com (L.Y.); duna1213@sina.com (N.D.); dangrr@xsyu.edu.cn (R.D.); binwang_1590@163.com (B.W.); xieyan70@163.com (Y.X.); 2 School of Marine Science and Technology, Northwestern Polytechnical University, Xi’an 710072, China; liuchangzan@mail.nwpu.edu.cn

**Keywords:** auxiliary sensor, transient electromagnetic techniques, borehole, nondestructive inspection

## Abstract

Transient electromagnetic (TEM) techniques are widely used in the field of geophysical prospecting. In borehole detection, the nondestructive inspection (NDI) of a metal pipe can be performed efficiently using the properties of eddy currents. However, with increasing concern for safety in oil and gas production, more than one string of pipe is used to protect wellbores, which complicates data interpretation. In this paper, an auxiliary sensor-based borehole TEM system for the NDI of multipipe strings is presented. On the basis of the characteristics of the borehole TEM model, we investigate the principle behind the NDI of multipipe strings using multiple time slices of induced electromotive force (EMF) in a single sensor. The results show that the detection performance of NDI is strongly influenced by eddy-current diffusion in the longitudinal direction. To solve this problem, we used time slices of the induced EMF in both the main and auxiliary sensors. The performance of the proposed system was verified by applying it to an oil well with a production casing and liner. Moreover, field experiments were conducted, and the results demonstrate the effectiveness of the proposed method.

## 1. Introduction

The transient electromagnetic (TEM) technique has gained much attention over the past several decades owing to its wide range of applications, such as mineral and petroleum geophysical exploration [1], hydrogeophysical surveys [2], and geotechnical and environmental investigation [3,4]. In the field of borehole detection, TEM systems enable the rapid acquisition of broad-frequency-range data related to the electrical and geometrical parameters of each borehole’s cylindrical layer [5,6,7]. This technique, which is also known as transient (pulsed) eddy-current testing [8,9,10,11], enables the highly effective nondestructive inspection (NDI) of downhole casings [12]. However, unlike the conventional NDI systems, oil and gas wells require more than one string of pipe, such as casing, tubing, and liner [13], to protect a wellbore against potential damage from byproducts, which makes NDI of the thickness of metal pipes more difficult in terms of data interpretation [14,15,16] because of the influence of additional pipe strings.

The problem associated with the NDI of the thickness of multilayer structures has been investigated extensively in numerous research fields [17,18,19]. In the case of borehole TEM systems, the early time data of the transient signal mainly correspond to the inner layers, whereas the late time data provide more information about the outer layers [15,16,17]. Consequently, the TEM data collected at different times correspond to each layer with different weights, and the response is assumed to be manifested as a convolution of the time decay signal, with each circular shell of metal with increasing radius corresponding to a “time window”, thereby approximately separating the receiving signal from different strings of pipe [15]. Similarly, using the longitudinal sensor, time slices as well as time windows of induced electromotive force (EMF) in the receiving coils are used as a feature to recognize defects in the NDI of casing through tubing [16]. The interpretation of the results obtained using the aforementioned methods based on the eddy-current diffusion property of TEM systems in the radial direction is straightforward. In the longitudinal direction, however, the eddy-current diffusion still exists, and the late time data also contain more information about the regions at increasing distances from the borehole axis; this information will strongly influence the effectiveness of the NDI of multipipe strings.

In this paper, we present an auxiliary sensor-based borehole TEM system for the NDI of the thickness of multipipe strings. On the basis of the characteristics of the borehole TEM signal model, an auxiliary sensor is used to improve the longitudinal resolution of the NDI of the thickness of multipipe strings, where induced EMFs in both the main and auxiliary sensors are utilized. We verify the performance of the proposed system by applying it to an oil–borehole TEM system used for the NDI of an oil well with a production casing and liner.

The rest of this paper is organized as follows. The borehole TEM signal model based on magnetic-core-coil sensors is presented in Section 2. The principle behind the NDI of multipipe strings, i.e., using multiple time slices of induced EMF in a single sensor, is discussed in Section 3. The diffusion property of the TEM sensor is analyzed and an auxiliary sensor-based borehole TEM system for the NDI of the thickness of multipipe strings is presented in Section 4. The experimental results are discussed in Section 5. Finally, we conclude the paper in Section 6.

## 2. Borehole TEM System Model

**Figure 1:**
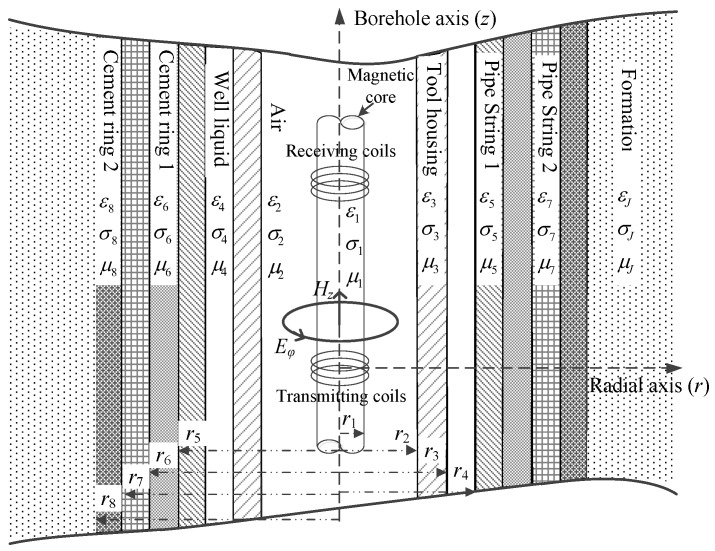
Cylindrically layered structures of a borehole.

The cylindrically layered structures of the borehole TEM system equipped with coaxial transmitting and receiving coils that are wound around a soft magnetic core are illustrated in Figure 1. The electrical and geometrical parameters of the *j*th layer are defined as (*μ_j_*, *ε_j_*, *σ_j_*) and *r_j_*, respectively. We consider the soft magnetic core to be the innermost layer. The transmitting and receiving coils are located in the second layer, with their number of turns given by *N*_T_ and *N*_R_, respectively. For all coils, the diameter is assumed to be sufficiently small and the source region is assumed to contain only the second layer. Moreover, all the other layers, such as the well liquid, casing, liner, cement, and formation, are regarded as source-free regions.

As shown in a previous study [20], the response of a TEM system in such a multicylindrically layered geometry consists of reflection and transmission components with both standing and outgoing waves. The vector potential **A** is introduced, and the homogeneous and inhomogeneous Helmholtz equations are given by

(1) $$\nabla^{2}A_{j}+k_{j}^{2}A_{j}=0\begin{array}{cc} & j\neq 2 \end{array},$$

(2) $$\nabla^{2}A_{2}+k_{2}^{2}A_{2}=-J_{e},$$

where *k_j_*^2^ *= μ_j_ε_j_ω*^2^ *− iμ_j_σ_j_ω*, and **J**_e_ denotes the electrical source. With the introduction of variables *x_j_* and *λ_j_* that satisfy *x_j_*^2^ = *λ_j_*^2^ − *k_j_*^2^, the vector potential **A** can be calculated by solving the Helmholtz equations. Considering the cylindrical symmetry model in Figure 1, the electric field is located in planes perpendicular to the borehole axis, and it has only the tangential component*.* Thereby, directional measurement cannot be achieved by using the proposed borehole TEM system. The electric field and the vertical component of the magnetic field of the *j*th layer with radius *r* and longitude (borehole axis) distance *z* can thus be obtained as [20]

(3) $$E_{\varphi j}\left(r,z\right)=-i\omega \mu_{j}M\int_{0}^{\infty }\left[\tau_{j}K_{1}(x_{j}r)I_{1}(x_{j}r_{1})+C_{j}I_{1}(x_{j}r)+D_{j}K_{1}(x_{j}r)\right]\cos\left(\lambda_{j}z\right)d\lambda_{j},$$

(4) $$H_{zj}\left(r,z\right)=\;M\int_{0}^{\infty }x_{j}\left[-\tau_{j}K_{0}(x_{j}r)I_{1}(x_{j}r_{1})+C_{j}I_{0}(x_{j}r)-D_{j}K_{0}(x_{j}r)\right]\cos\left(\lambda_{j}z\right)d\lambda_{j},$$

with *M* = *I*_T_*r*_1_/π, *τ*_2_ = 1, and *τ_j_*_≠2_ = 0, where *I*_T_ denotes the transmitting current; *I*_0_(·), *I*_1_(·), *K*_0_(·), and *K*_1_(·) are the first and second type of modified Bessel functions of order zero and one, respectively, and *C_j_* and *D_j_* denote the reflection and transmission coefficients, respectively, which are related to the geometrical and electrical parameters of all layers, and can be calculated using the boundary conditions. Then, the induced EMF in the receiving coils can then be calculated by

(5) $$U(\omega )=-i\omega \mu_{1}\sum_{m=0}^{N_{T}-1}\sum_{n=0}^{N_{R}-1}\int_{0}^{r_{1}}H_{z1}(r,z_{mn})\cdot 2\pi rdr,$$

where *z_mn_* denotes the distance between the *m*th turn of transmitting coils and the *n*th turn of receiving coils along the borehole axis. Given a ramp signal with a turn-off time of *t*_0_, the induced EMF *U*(*t*) can be obtained by converting Equation (5) into the time domain; using the Gaver–Stehfest inverse Laplace transform as an example [20], we obtain

(6) $$U(t)=\frac{\mu_{1}\pi r_{1}^{2}}{t_{0}}\frac{\ln2}{t}\sum_{q=1}^{12}K_{q}\frac{1}{s_{q}}(e^{-s_{q}t_{0}}-1)\int_{0}^{r_{1}}H_{z}{}_{1}\cdot 2\pi rdr,$$

where *s_q_* = *q*ln2/*t* and *K_q_* denotes the integral coefficient of the Gaver–Stehfest inverse Laplace transform. The tool housing is fixed, and the conductivities of the cement ring, formation, and fluids are much smaller than that of the metal pipes, whose thickness can be estimated from *U(t)* by ignoring the effect of the other layers. Moreover, if only one pipe string exists, a single time slice of the induced EMF, whose amplitude monotonically increases with the thickness of the metal pipe, can be used to describe the relationship between *U*(*t*) and thickness [5,16]. However, when more than one string of metal pipes is present in oil and gas wells, the coupling of the response from the additional pipe strings will strongly influence the interpretation of the NDI, where additional time slices of the induced EMF in the receiving coils must be used to interpret the logging data.

## 3. NDI of Multipipe Strings

As shown in Section 2, the presence of more than one pipe string makes NDI data interpretation more difficult, because the TEM response is strongly influenced by additional pipe strings. In this section, we investigate the principle behind the NDI of multipipe strings using multiple time slices of induced EMF in a single sensor. Considering the boundary conditions of multilayered cylindrical structures that the tangential component of the electric field and the tangential component of the magnetic field are continuous across the interface in the case of *r* = *r_j_*, we have *E_φ,j_* = *E_φ,j+1_* and *H_z,j_* = *H_z,j+1_* [20]. Thereby, the relationship between the coefficients *C_j_* and *D_j_* of each layer can be written as

(7) $$\left[\begin{array}{l} C_{j}\\ D_{j} \end{array}\right]=-\frac{r_{j}}{\mu_{j}}P_{j}\left[\begin{array}{l} C_{j+1}\\ D_{j+1} \end{array}\right]-\tau_{j+1}\frac{r_{j}}{\mu_{j}}\left[\begin{array}{c} P_{j}\left(1,1\right)\\ P_{j}\left(2,1\right) \end{array}\right]K_{1}(x_{j+1}r_{1})+\left[\begin{array}{c} 0\\ -\tau_{j}I_{1}(x_{j}r_{1}) \end{array}\right],$$

where **P***_j_* is the transfer matrix, with each element expressed as

(8) $$P_{j}(1,1)=-\mu_{j+1}x_{j}K_{0}(x_{j}r_{j})I_{1}(x_{j+1}r_{j})-\mu_{j}x_{j+1}K_{1}(x_{j}r_{j})I_{0}(x_{j+1}r_{j}),$$

(9) $$P_{j}(1,2)=-\mu_{j+1}x_{j}K_{0}(x_{j}r_{j})K_{1}(x_{j+1}r_{j})+\mu_{j}x_{j+1}K_{1}(x_{j}r_{j})K_{0}(x_{j+1}r_{j}),$$

(10) $$P_{j}(2,1)=-\mu_{j+1}x_{j}I_{0}(x_{j}r_{j})I_{1}(x_{j+1}r_{j})+\mu_{j}x_{j+1}I_{1}(x_{j}r_{j})I_{0}(x_{j+1}r_{j}),$$

(11) $$P_{j}(2,2)=-\mu_{j+1}x_{j}I_{0}(x_{j}r_{j})K_{1}(x_{j+1}r_{j})-\mu_{j}x_{j+1}I_{1}(x_{j}r_{j})K_{0}(x_{j+1}r_{j}).$$

In Equation (7), *C_J_* and *D*_1_ have been proved to be zero because of the absence of transmission and reflection of the innermost and outermost layers, respectively. Then, *C*_1_ can be derived using the relationship described in Equation (7), as

(12) $$C_{1}=-\frac{r_{1}}{\mu_{1}}\left(T\frac{P(1,2)}{P(2,2)}-V\right)$$

with

(13) $$P=P_{1}\cdot P_{2}\cdot \;\cdots \;\cdot P_{J},$$

(14) $$T=P_{1}\left(2,1\right)K_{1}(x_{2}r_{1})-\tau_{j}P_{1}\left(2,2\right)I_{1}(x_{1}r_{1}),$$

(15) $$V=P_{1}\left(1,1\right)K_{1}(x_{2}r_{1})-\tau_{j}P_{1}\left(1,2\right)I_{1}(x_{1}r_{1}).$$

Note that *C_1_* is related to the electrical and geometrical parameters of the multicylindrical layers and to the diffusion time. To measure the thickness of metal pipes, we assume that the electrical parameters of all layers and the outer radius of the metal pipes are fixed. Thus, for each sampling time, the unknown variables remaining in Equation (12) are the thicknesses of the multipipe strings. Obviously, if only one string of pipes is involved, Equation (12) will contain just one unknown variable and can be solved using a single time slice of induced EMF. Correspondingly, when more than one string of pipe is utilized, multiple time slices of induced EMF must be employed to solve Equation (12). Taking two strings of pipes as an example, the relationships between the induced EMF and the thickness of the two pipes at an early time (20 ms) and a late time (50 ms) are shown as follows, where the simulation parameters are set as follows: *r*_1_ = 12 mm, *r*_2_ = 17.5 mm, *r*_3_ = 21 mm, *r*_4_ = 54.31 mm, *r*_6_ = 87.31 mm, *r*_8_ = 120.65 mm, *N*_T_ = 95, *N*_R_ = 980, *I*_T_ = 0.5 A, and *t*_0_ = 30 μs.

**Figure 2:**
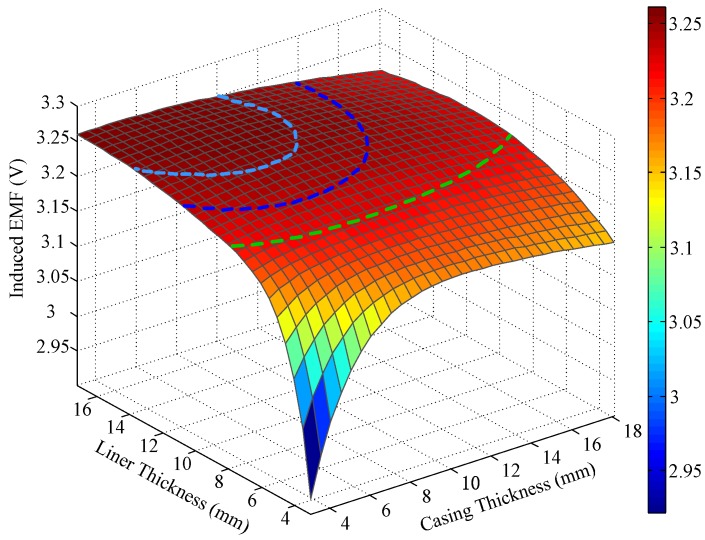
Induced electromotive force (EMF) in receiving coils at 20 ms.

**Figure 3:**
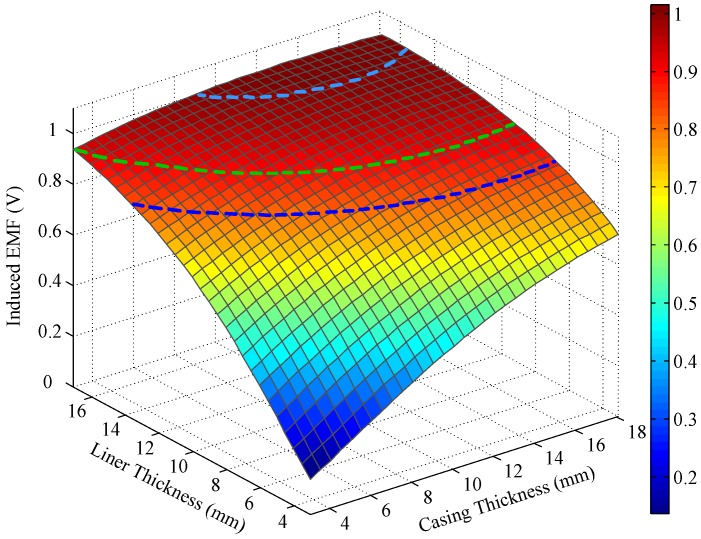
Induced EMF in receiving coils at 50 ms.

**Figure 4:**
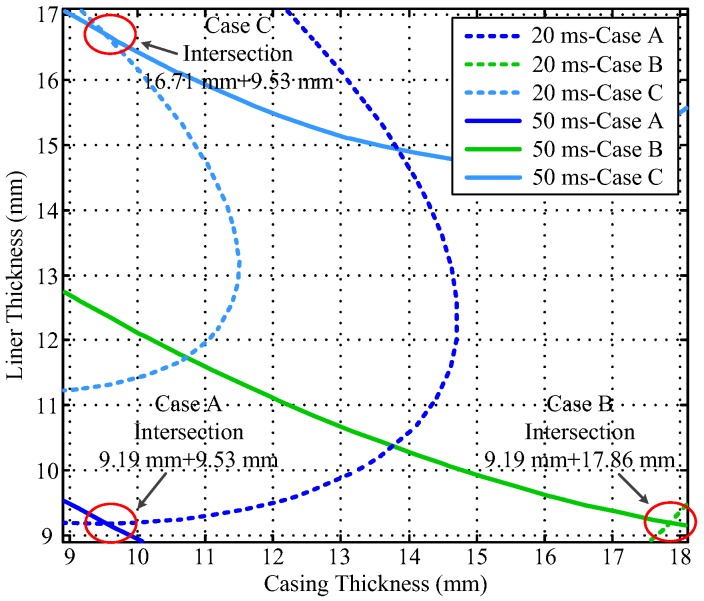
Intersection of induced EMF contour curves at 20 and 50 ms.

Notably, the liner and casing in Figure 2 and Figure 3 represent the first pipe string and the second pipe string as shown in Figure 1, where a liner is a pipe string that does not extend to the surface, being hung from a liner hanger set inside of the previous pipe string [13]. Corresponding to Figure 1, the inner radiuses of the two pipes (*r*_4_ and *r*_6_) are assumed to be fixed, which can be obtained by the prior wellbore information, so that the thicknesses of the two pipe strings are related to the outer radiuses of the two pipes (*r*_5_ and *r*_7_), and can be calculated by *r*_5_ − *r*_4_ and *r*_7_ − *r*_6_, respectively. In Figure 2 and Figure 3, the thicknesses of the two pipe strings are changed within a range to show the characteristics of the borehole TEM system. Moreover, the three dashed lines denote the contour curves of the induced EMF value with respect to the three combinations of the thicknesses of two pipes (termed Cases A, B, and C) at different times. We find that the induced EMF of a single time slice corresponds to numerous solutions, which obviously makes Equation (12) unsolvable. Nevertheless, by employing the eddy-current diffusion property of the TEM system, where the data at different times correspond to the information of each layer with different weights [15,16,17], we can solve Equation (12) through the association of multiple time slices. Projecting the contour curves onto the casing–liner thickness plane, we describe the characteristics of the induced EMF contour curves as shown in Figure 4.

**Figure 5:**
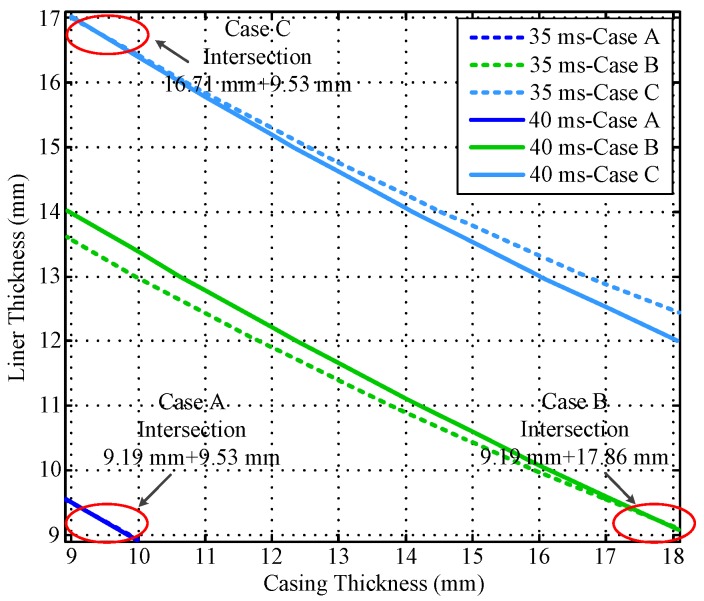
Intersection of induced EMF contour curves at 35 and 40 ms.

Figure 4 shows that although each single time slice of the induced EMF cannot have a unique solution for two metal pipe thicknesses, their projections can intersect at one point for each case. Since each induced EMF contour curve represents a combination of solutions of Equation (12) at different times, the intersection point can be regarded as the unique solution of Equation (12) that satisfies the two curves simultaneously. Thereby, on the basis of the eddy-current diffusion property, two time slices of the induced EMF can be used to achieve the NDI of two pipe strings. Specifically, when the two time slices have been selected, the two projected contour curves according to the corresponding EMF values are then obtained with the simulation results approximately. As a result, the intersection of the two curves can be used to interpret the thickness of the two pipe strings. Theoretically, the combinations of the time slices are not unique. However, the similarity of the two EMF curves for each case is inversely proportional to the difference between the two observation times. Thereby, although different combinations of the time slices may lead to different inspection performance, as long as the contour curves are not too similar, lots of combinations of time slices could be employed to obtain almost the same results. In this paper, the effectiveness of the principle of the NDI of multipipe strings is demonstrated by using the two time slices at 20 and 50 ms as an example shown in Figure 4 to ensure that the two EMF curves are sufficiently far apart to provide adequate noise suppression. By contrast, if the two time slices were too close (e.g., 35 ms and 40 ms) as shown in Figure 5, the projected contour curves will become almost parallel, which will not only make the intersection very difficult to distinguish, but also influence the ability of noise suppression.

In practice, when the difference between the two time slices is less than 10 ms, the intense noise due to a poor downhole environment will make the two curves as well as the intersection point difficult to distinguish and recognize. Notably, the detection performance can be further improved through the optimization of the chosen time slices; this will be investigated in our future work. Furthermore, this method can also be extended to solve the problem of the NDI of multipipe strings, where the dimensions of induced EMF contour curves should be correspondingly increased. Unfortunately, in borehole detection, the late time data of the TEM response contain more information corresponding to increasing distance not only in the radial direction but also in the longitudinal direction, which will strongly affect the detection performance of the borehole TEM system for the NDI of multipipe strings.

## 4. Auxiliary Sensor-Based NDI of Multipipe Strings

**Figure 6:**
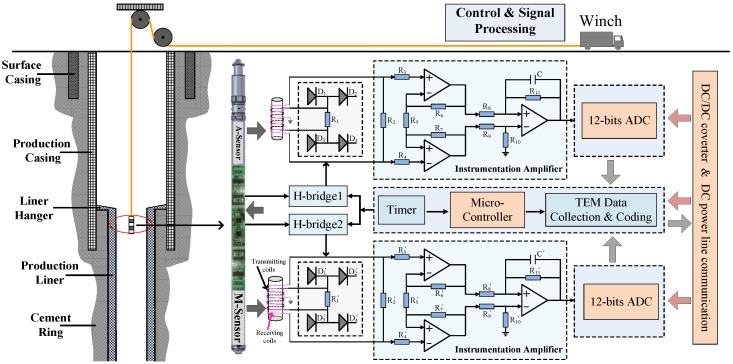
Auxiliary sensor-based borehole transient electromagnetic (TEM) system in a multistring oil well. DC, direct current. ADC, analog-to-digital converter.

On the basis of the model for the signal of the borehole TEM system for NDI, we showed that the thickness of two pipe strings can be estimated by detecting two time slices of induced EMF in a single sensor. Now, we show how the auxiliary sensor-based borehole TEM system (Figure 6) can be used to improve the detection performance of the NDI of multipipe strings. The measurement tool shown in Figure 6 comprises two sensors and their measurement circuits, which are fixed in a waterproof tool housing. Each sensor consists of transmitting and receiving coils wound around a magnetic core with a radius of 6 mm (auxiliary sensor, termed as A-sensor) and 12 mm (main sensor, termed as M-sensor), respectively, where the main sensor is the same one discussed in Section 3. The number of turns of the two sensors is the same.

The measurement circuits mainly consist of a direct current (DC)–DC converter, two transmission waveform generators (H-bridge), a microcontroller, two analog-to-digital converters (ADC), and two instrumentation amplifiers. The assembled circuits have a working temperature of at least 150 °C for deep borehole inspection. The data collected from the sensors are transmitted to the surface system via DC power line communication in real time. In this paper, we use the proposed borehole TEM system for the NDI of two strings of the pipe (production casing and liner).

**Figure 7:**
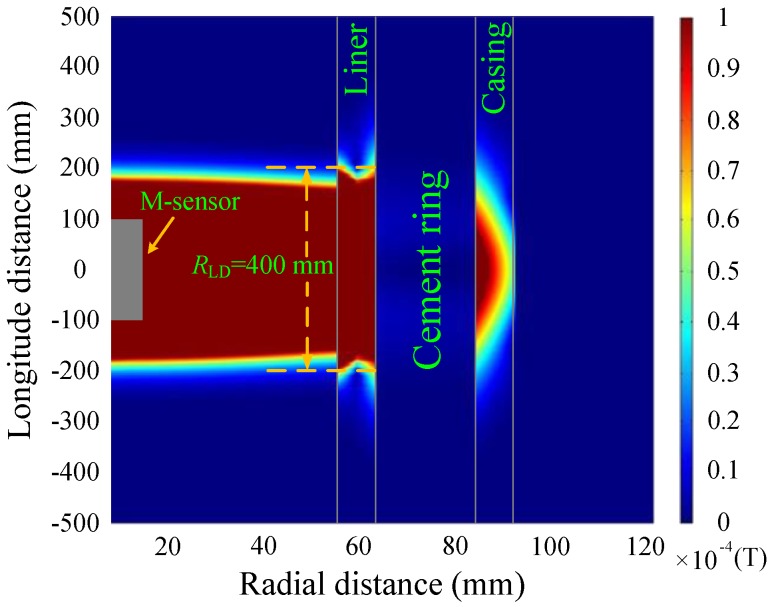
Eddy-current field of the main sensor at 20 ms.

**Figure 8:**
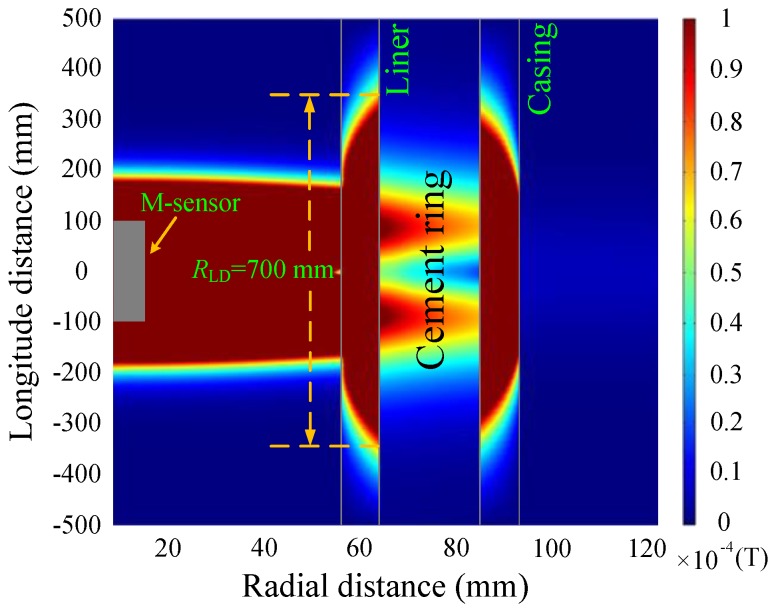
Eddy-current field of the main sensor at 50 ms.

Figure 7 and Figure 8 show the simulations of the eddy-current fields, which are obtained by converting the vertical component of the magnetic field shown in Equation (4) into the time domain, and are described by magnetic flux density with the relative permeability of each layer, where *R*_LD_ denotes the longitudinal diffusion range of the sensor. It is noted that since the transmitting multi-turn coils are not a point source, the total response should be calculated by summing the response of each single turn coil with its corresponding *z* coordinates.

**Figure 9:**
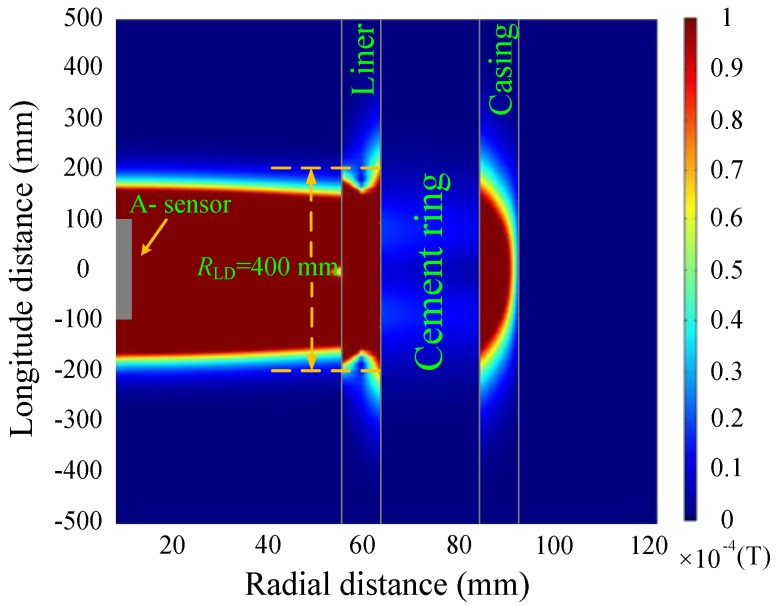
Eddy-current field of the auxiliary sensor at 30 ms.

As shown in Figure 7 and Figure 8, the diffusion of the eddy-current fields occurs not only in the radial direction but also in the longitudinal direction. In the radial direction, the influence of the casing on the magnetic field at a late time (50 ms) is stronger than that at an early time (20 ms). In the longitudinal direction, the diffusion range obviously increases with observation time, where the two time slices used for NDI correspond to different effective detection ranges of approximately 400 and 700 mm. Although the eddy-current diffusion property in the radial direction can help solve the problem associated with the NDI of multipipe strings, it will also result in a substantial decrease in both the longitudinal resolution and the accuracy for detecting changes in thickness; this may lead to a model mismatch for the borehole TEM system because of the thickness inhomogeneity of the metal pipe, which means the thickness of the metal pipe is inhomogeneous along the borehole axis (or longitudinal direction), i.e., the thickness changes due to the existence of the collars or pipe damage. As an alternative, we chose to use time slices of induced EMF in both the main sensor and the auxiliary sensor to avoid an excessively large longitudinal diffusion range (*R*_LD_)_._ The eddy-current field of the auxiliary sensor at 30 ms is shown in Figure 9.

**Figure 10:**
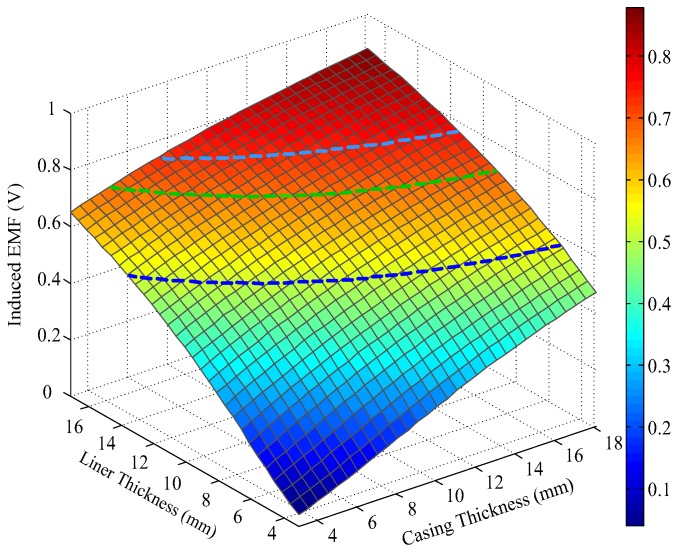
Induced EMF in the auxiliary sensor at 30 ms.

**Figure 11:**
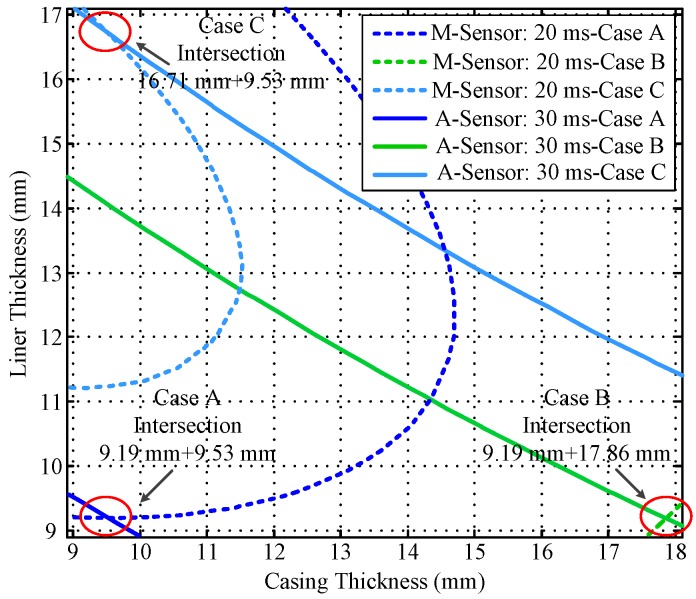
Intersection of the induced EMF curves using the auxiliary sensor.

A comparison of Figure 7 and Figure 9 reveals that the auxiliary sensor at 30 ms has almost the same longitudinal diffusion range as the main sensor at 20 ms, whereas their radial diffusion properties differ. On the one hand, because of their different radial properties, the two metal pipes differently influence the response of the magnetic field; thus, the principle behind the NDI of multipipe strings, discussed in Section 3, can be used to solve Equation (12) with two unknown thicknesses. On the other hand, because the two time slices have approximately the same low *R*_LD_ value, the mismatch of the borehole TEM signal model at different times will be reduced, and the longitudinal resolution as well as the detection accuracy will be improved. Similar to Figure 2, Figure 3 and Figure 4, Figure 10 and Figure 11 show the induced EMF of the two sensors for the three cases and their corresponding intersections (solutions) for two thicknesses, respectively.

A comparison of Figure 2, Figure 3, and Figure 10 reveals that the induced EMF curved surface of the auxiliary sensor at 30 ms differs from that of the main sensor at 20 ms, but is similar to that of the main sensor at 50 ms; thus, the auxiliary sensor method can achieve noise suppression similar to that achieved using the single-sensor NDI method. This capability stems from the auxiliary sensor having a smaller radius compared with the main sensor, thereby causing its time decay to be much shorter; thus, the observation time of 30 ms for the auxiliary sensor is already a late time, similar to the time slice of 50 ms for the main sensor.

Furthermore, Figure 11 shows that the projection of the induced EMF contour curves of the two sensors at 20 and 30 ms can still intersect at one point for each case. Thereby, the NDI of two strings of pipes can also be achieved using the auxiliary sensor, with better detection performance of longitudinal resolution and detection accuracy.

## 5. Field Experiments

### 5.1. Experimental Results

The validity of the auxiliary sensor method for the NDI of multipipe strings was confirmed by field experiments conducted at the Yaerxia oil production plant Yumen Oilfield, China. The experiments were conducted in a production oil well with two strings of pipes comprising a production casing and liner. In our experiment, *r*_1_, *r*_2_, *r*_3_, *r*_4_, *r*_6_, and *r*_8_ have the same value as the simulation in the above sections. The parameters of the two sensors are shown in Table 1.

In their actual sizes, the two types of metal pipe in our paper have thicknesses of 9.19 and 9.53 mm, respectively, where each liner or casing is connected by a collar with a thickness of approximately 7.52 mm (liner) and 8.33 mm (casing); thus, the corresponding thickness in the collar becomes 16.71 mm for the liner and 17.86 mm for the casing approximately as shown in [13]. Furthermore, the inner radius in the collar is the same as the pipe, while their outer radiuses are different. In this paper, the liner and casing collars that can be validated with prior and authentic knowledge are analyzed as examples to evaluate the effectiveness of the proposed method.

**Figure 12:**
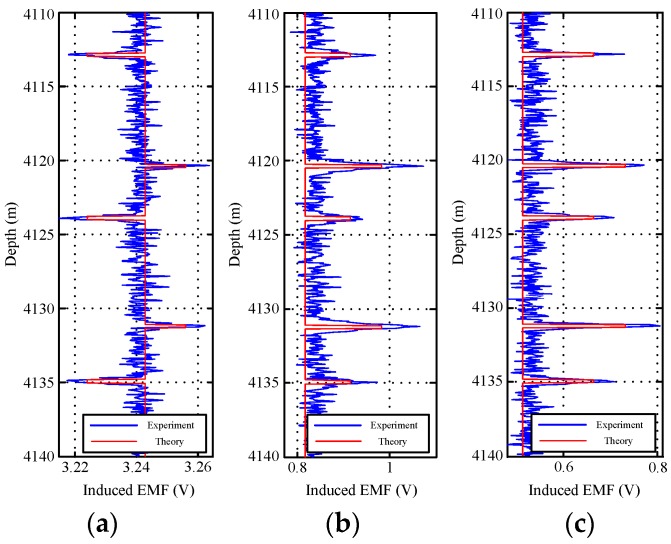
Induced EMF of the two sensors at different times ranging from 4110 to 4140 m: (**a**) main sensor at 20 ms; (**b**) main sensor at 50 ms; and (**c**) auxiliary sensor at 30 ms.

**Figure 13:**
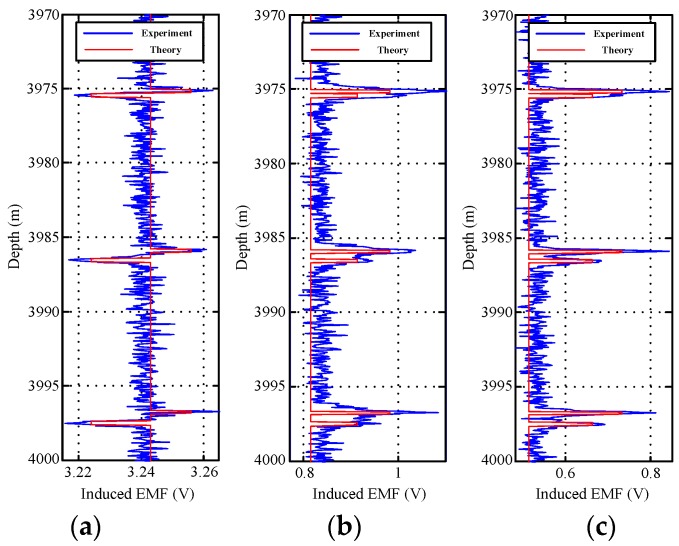
Induced EMF of the two sensors at different times ranging from 3970 to 4000 m: (**a**) main sensor at 20 ms; (**b**) main sensor at 50 ms; and (**c**) auxiliary sensor at 30 ms.

**Figure 14:**
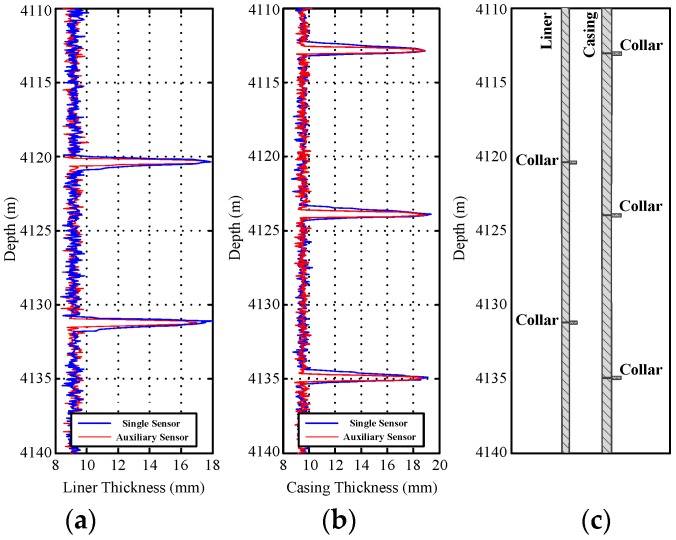
Interpreted thickness using the NDI of two-pipe strings ranging from 4110 to 4140 m: (**a**) liner thickness; (**b**) casing thickness; and (**c**) pipe string structure.

**Figure 15:**
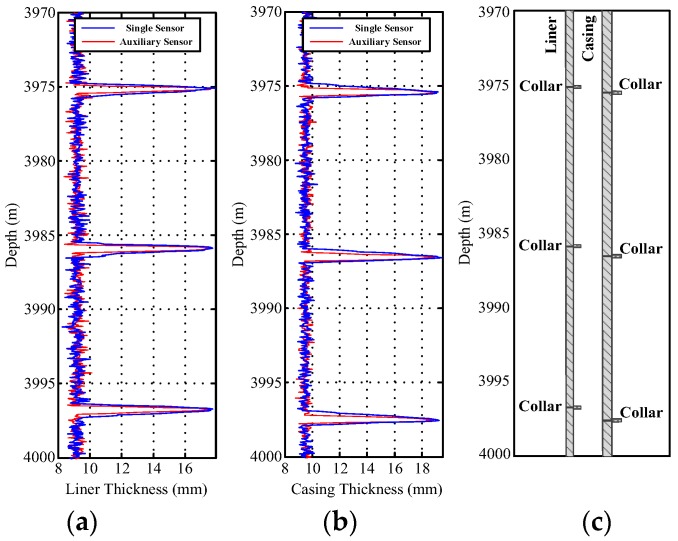
Interpreted thickness using the NDI of two-pipe strings ranging from 3970 to 4000 m: (**a**) liner thickness; (**b**) casing thickness; and (**c**) pipe string structure.

Figure 12 and Figure 13 show the field experiment results and theoretical values of the induced EMFs from 4110 to 4140 m and from 3970 to 4000 m, respectively. Similar to Figure 4 and Figure 11, the experiment data in Figure 12 and Figure 13 can be used to interpret the thickness of the two pipe strings on the basis of the NDI of the thickness of multipipe strings. Additionally, the interpreted thicknesses with the proposed auxiliary sensor method and the single-sensor method for the NDI of multipipe strings are shown in Figure 14 and Figure 15, where the corresponding pipe string structures are also illustrated. It can be observed from Figure 12, Figure 13, Figure 14 and Figure 15 that the 'peaks' are corresponding to the collars, which means that the experiment data are consistent with the pipe string structures, thereby demonstrating the feasibility of the proposed borehole TEM system. Also, as evident in the figures, all of the collars (experimental results) shown in Figure 12b and Figure 13b appear much wider than those shown in Figure 12a,c and Figure 13a,c; this indicates the poorer resolution performance of NDI in the borehole axis when the induced EMF in the main sensor at 50 ms is used. Moreover, at several distances between the nearby liner and casing collars (termed *D*_NC_), the detected signals of the main sensor at a late time also do not perform well to distinguish nearby collars. Thereby, although multiple time slices of the induced EMF from a single sensor can be used to interpret the thickness of two-pipe strings, the longitudinal resolution may be influenced by large *R*_LD_ at a late time.

### 5.2. Analysis and Discussion

Using the experimental results, we demonstrated the effectiveness of the proposed borehole TEM system for the NDI of multipipe strings. Note that smaller *R*_LD_ values indicate that an improved detection performance of NDI can be achieved as shown in Section 4. In this section, on the basis of the interpreted thicknesses, we will discuss the longitudinal resolution of the auxiliary sensor-based method by analyzing the measured width of the casing or liner collars along the borehole axis, which is related with the thickness of the pipes.

**Figure 16:**
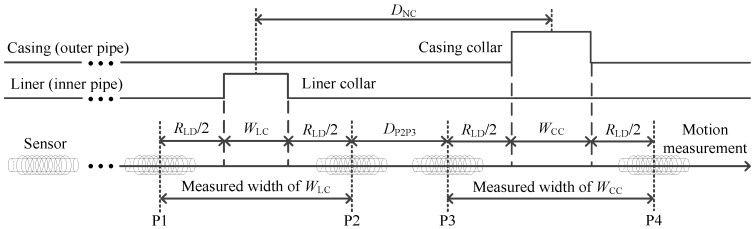
Relationships among the longitudinal detection range *R*_LD_, the distance between nearby liner and casing collars *D*_NC_, and the measured width.

**Figure 17:**
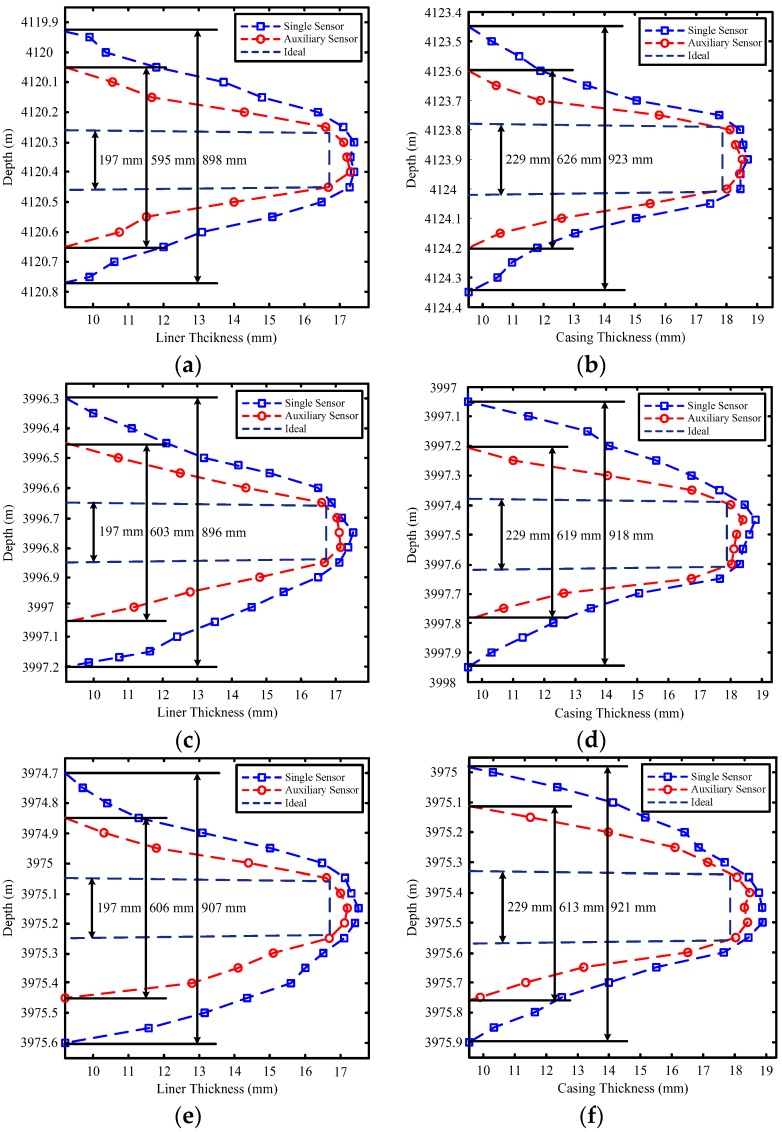
Thickness of the liner and casing with collars: (**a**) liner collar at 4120.36 m; (**b**) casing collar at 4123.9 m; (**c**) liner collar at 3996.75 m; (**d**) casing collar at 3997.5 m; (**e**) liner collar at 3975.15 m; and (**f**) casing collar at 3975.45 m.

In Figure 16, we describe the relationships between *R*_LD_, *D*_NC_, and the measured width, where *W*_LC_ and *W*_CC_ denote the actual width of the liner and casing collar, respectively. P1, P2, P3, and P4 denote the four particular observation locations. Note that the liner and casing collars can be detected only in a limited range between P1 and P2 and between P3 and P4, respectively. Thus, the measured width of the liner and casing collars can be represented by the distance between P1 and P2 and between P3 and P4, respectively. We then define the *D*_NC_ as follows:

(16) $$D_{\mathrm{NC}}=\frac{1}{2}W_{\mathrm{LC}}+\frac{1}{2}W_{\mathrm{CC}}+R_{\mathrm{LD}}+D_{P2P3}$$

where *D*_P2P3_ represents the distance between P2 and P3. As shown in Figure 16, when *D*_P2P3_ > 0, the two sensors will not be influenced by each other. However, when the two collars are too close (*D*_P2P3_ < 0), the two nearby collars will strongly affect each other, resulting in a substantial decrease in the detection accuracy of the NDI of the two strings of the pipe. Taking three typical couples of the nearby collars in Figure 12 and Figure 13 as examples, we describe the inversed thickness using the NDI method described in Section 3; the results are presented in Figure 17.

Figure 17 compares the inversed liner and casing thicknesses obtained using the proposed auxiliary sensor method and the single-sensor method as well as those obtained in the ideal case. The actual widths of the liner and casing collars are 197 and 229 mm, respectively. In Figure 17, the inversed widths obtained using the two sensor methods are all expanded when compared with the ideal case; this is caused by the model mismatch for the multicylindrical borehole TEM system owing to the large diffusion range of the two sensors for detecting the change in thickness. For comparison, as shown in Figure 17, for all cases, the inversed widths of the liner and casing collars obtained using the auxiliary sensor method are approximately 600 mm, whereas the inversed widths obtained using the single-sensor method are approximately 900 mm. Notably, the inversed width has almost the same value as the larger measured width of the two time slices used for the NDI of multipipe strings, and can be calculated by summing the actual width of the collar and the *R*_LD_ illustrated in Figure 16. Moreover, as shown in Figure 17c,d, when *D*_NC_ = 750 mm, which means *D*_P2P3_ < 0 for the M-sensor at a late time, the accuracy of the single-sensor method is drastically influenced by the two nearby collars, where the inversed thickness curves deform at the nearby collar side. However, the auxiliary sensor method shows almost the same detection performance in Figure 17a,b because *D*_P2P3_ > 0 for the A-sensor. In Figure 17e,f, with a *D*_NC_ of approximately 300 mm, where *D*_P2P3_ < 0 for both sensors, although the performance of the proposed method decreases, it remains better than that of the single-sensor method.

**Figure 18:**
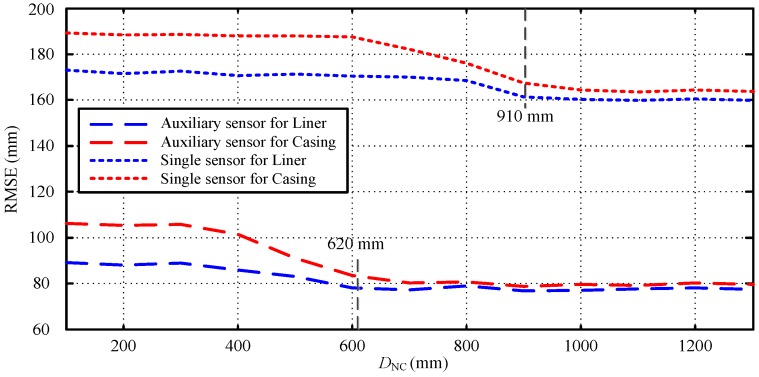
Root-mean-square error (RMSE) of each collar versus *D*_NC_.

Without loss of generality, we employ the root-mean-square error (RMSE) of each collar versus *D*_NC_ to illustrate the detection accuracy observed in Figure 18. The RMSE is defined as

(17) $$\mathrm{RMSE}=\sqrt{\frac{1}{L}\sum_{l=0}^{L-1}{\left(T_{i,l}-T_{a,l}\right)}^{2}},$$

where *L* is the total number of the observation points for each collar, and *T_i,l_* and *T_a,l_* denote the inversed and actual thickness of each observation point, respectively. We observe in Figure 18 that for all values of *D*_NC_, the proposed method performs a much better NDI of two strings of pipes than the single-sensor method, and the inner pipe always shows a better NDI performance. Moreover, the RMSE of the proposed method is shown to not become larger until the *D*_NC_ is smaller than approximately 620 mm; however, the performance of the single-sensor method becomes unreliable even for *D*_NC_ values smaller than approximately 910 mm.

## 6. Conclusions

An auxiliary sensor-based borehole TEM system was proposed to improve the longitudinal resolution of the NDI of multipipe strings. We illustrated the principle behind the NDI of multipipe strings using multiple time slices of induced EMF in a single sensor. The results show that the detection performance of NDI was strongly influenced by the eddy-current diffusion property along the borehole axis. Moreover, by employing the time slices of the induced EMF in the auxiliary sensor, the thickness of two metal pipes could be inversed more accurately. Field experiments for an oil–borehole liner and casing inspection at the Yumen Oilfield demonstrated the effectiveness of the proposed system.

## Figures and Tables

**Table 1:** Sensor parameters for the nondestructive inspection (NDI) of multipipe strings.

Parameter	Value
Radius of the main sensor	12 mm
Radius of the auxiliary sensor	6 mm
Number of transmitting coil turns	95
Number of receiving coil turns	980
Wire diameter of the transmitting coils	0.46 mm
Wire diameter of the receiving coils	0.18 mm
Resistance of the transmitting coils of the A-sensor	1.57 Ω
Resistance of the receiving coils of the A-sensor	52.8 Ω
Resistance of the transmitting coils of the M-sensor	3.12 Ω
Resistance of the receiving coils of the M-sensor	105.1 Ω
